# BTK: sensing pathogenic nucleic acids

**DOI:** 10.18632/oncotarget.4778

**Published:** 2015-07-01

**Authors:** Koon-Guan Lee, Kong-Peng Lam

**Affiliations:** Bioprocessing Technology Institute, Singapore, Department of Microbiology, Physiology and Paediatrics, Yong Loo Lin School of Medicine, National University of Singapore, Singapore and School of Biological Science, Nanyang Technological University, Singapore

**Keywords:** Immunology and Microbiology Section, Immune response, Immunity

We are under constant threats from pathogens. A failure to initiate an appropriate immune response will lead to an immunocompromised state. Our innate immune system could sense foreign nucleic acids from parasites, viruses, fungi and bacteria. This is achieved through pattern recognition receptors (PRRs) on innate cells such as macrophages that recognise different pathogen-associated molecular patterns including those found on foreign nucleic acids.

Viral double-stranded RNA (dsRNA) in particular are recognised by PRRs such as endosomal Toll-like receptor (TLR)-3 and cytosolic RIG-I-like receptors (RLRs), RIG-I. Long dsRNA binds to TLR3 leading to receptor dimerization and phosphorylation of two tyrosine residues in its intracellular domain, Tyr759 and Tyr858 [1]. TRIF adaptor downstream of TLR3 subsequently recruits TBK1 to activate Interferon regulatory factor (IRF) 3. PI3K is reported to bind to phosphorylated TLR3 at Tyr759 [1] and activate downstream AKT for signaling to IRF3 as well. In addition, RIP1 binds to the C-terminus of TRIF to activate NFκB signaling. Finally, MAP kinases are phosphorylated downstream of TRIF for AP-1 signaling and together with NFκb and IRF3 transcription factors, cooperatively activate Type 1 IFN production, particularly IFN-β, critical for antiviral response [2].

Since TLR3 signaling is complex, we hypothesized that more molecules could be involved. Bruton's tyrosine kinase (BTK) is critical for B cell development and mutations in BTK leads to *X-linked agammaglobulinaemia* (XLA) in humans and *X-linked immunodeficiency* in mice [3]. XLA patients were also observed to develop recurrent bacterial and viral infections suggesting a possible role for BTK in innate immunity. Indeed BTK were found to be activated by several TLRs that signal through the adaptor MyD88 [4]. TLR3 signaling is unique as it strictly uses only the TRIF adaptor, and hence presented a good system to examine if BTK plays a role in TRIF-signaling [5]. We found D-galactosamine sensitised BTK knockout mice to survive better than wildtype mice when challenged with poly(I:C). *Ex vivo* experiments using macrophages from wildtype and *btk*_−/−_ mice stimulated with naked poly(I:C) revealed that the production of inflammatory cytokines and IFN-β were defective in the absence of BTK. To corroborate this finding, we further infected wildtype and *btk*_−/−_ macrophages with dengue viruses. Consistent with prior observations of the requirement of BTK in TRIF signaling for Type 1 IFN production, dengue virus infected *btk*_−/−_ macrophages were found to have defective IFN-β mRNA upregulation and unable to clear dengue virus infection.

To gain insight into BTK's role in TLR3 signaling, we made mutant constructs resulting in either constitutive-active (CA) or kinase-dead (KD) forms of BTK. In over expression studies using HEK293 cells together with TLR3, we observed that active BTK phosphorylates Tyr759 residues of TLR3 [5]. Thus BTK plays a critical role in TLR signaling.

DNA sensors have recently emerged as new classes of PRRs that also signal Type 1 IFN production. Some of these receptors include IFI16, DAI, LRRFIP1, DDX41 and cGAS and they recognise pathogenic dsDNA enriched in AT sequences [6]. It is now established that some of these intracellular sensors signal via the adaptor STING. In our recent study [7], we examined the possibility that BTK might have a role in STING signaling. We challenged *btk*_−/−_ mice with several agents that triggered STING signaling such as dAdT, DMXAA and malaria DNA and found that the production of IFN-β was defective with all stimulants studied. We also observed not only increased susceptibility but also higher parasitemia development in BTK knockout mice challenged with *plasmodium yoelii* compared to wildtype controls. As malaria dsDNA is rich in AT sequences, we postulated that BTK may be required in the innate sensing of intracellular DNA. We examined further and confirmed that BTK could bind STING to activate Type 1 IFN signaling. Our biochemical and mass-spectrometry experimental approaches further uncover BTK's role in activating the DNA sensor DDX41. BTK was found to phosphorylate Tyr414 of the DDX41 helicase to activate its recognition of dsDNA and subsequent binding to STING. This further triggers STING-dependent TBK1 phosphorylation and activation of IRF3 to initiate IFN-β mRNA synthesis.

In summary, we identified a critical role for BTK in innate immunity. BTK phosphorylates TLR3 and DDX41 that are important for recognizing intracellular nucleic acids including double-stranded RNA and DNA. We further speculate that BTK could activate even more innate immune receptors. Drug discovery in future could be aimed at identifying chemicals that either dampen or boost BTK's activity and therefore modulate chronic inflammation, infectious diseasesand cancer.

## References

[1] Sarkar SN (2004). Nature Structural & Molecular Biology.

[2] Kim T (2000). Journal of Biological Chemistry.

[3] Lindvall JM (2005). Immunological Reviews.

[4] Doyle SL (2007). Journal of Biological Chemistry.

[5] Lee K-G (2012). Proceedings of the National Academy of Sciences.

[6] Paludan Søren R (2013). Immunity.

[7] Lee K-G (2015). Cell Reports.

